# Controlling the Growth of the Skin Commensal Staphylococcus epidermidis Using d-Alanine Auxotrophy

**DOI:** 10.1128/mSphere.00360-20

**Published:** 2020-06-10

**Authors:** David Dodds, Jeffrey L. Bose, Ming-De Deng, Gilles R. Dubé, Trudy H. Grossman, Alaina Kaiser, Kashmira Kulkarni, Roger Leger, Sara Mootien-Boyd, Azim Munivar, Julia Oh, Matthew Pestrak, Komal Rajpura, Alexander P. Tikhonov, Traci Turecek, Travis Whitfill

**Affiliations:** a Azitra Inc., Farmington, Connecticut, USA; b Department of Microbiology, Molecular Genetics and Immunology, University of Kansas Medical Center, Kansas City, Kansas, USA; c Bio-Technical Resources, Manitowoc, Wisconsin, USA; d Department of Psychiatry, Yale-New Haven Hospital, New Haven, Connecticut, USA; e Jackson Laboratory for Genomic Medicine, Farmington, Connecticut, USA; f Department of Pediatrics, Yale University, New Haven, Connecticut, USA; University of Nebraska Medical Center

**Keywords:** microbiome, skin, engineering, genetics, therapeutics, live biotherapeutic products, dermatology, synthetic biology

## Abstract

Using live microbes as therapeutic candidates is a strategy that has gained traction across multiple therapeutic areas. In the skin, commensal microorganisms play a crucial role in maintaining skin barrier function, homeostasis, and cutaneous immunity. Alterations of the homeostatic skin microbiome are associated with a number of skin diseases. Here, we present the design of an engineered commensal organism, Staphylococcus epidermidis, for use as a live biotherapeutic product (LBP) candidate for skin diseases. The development of novel bacterial strains whose growth can be controlled without the use of antibiotics or genetic elements conferring antibiotic resistance enables modulation of therapeutic exposure and improves safety. We therefore constructed an auxotrophic strain of S. epidermidis that requires exogenously supplied d-alanine. The S. epidermidis NRRL B-4268 Δ*alr1* Δ*alr2* Δ*dat* strain (SE_ΔΔΔ_) contains deletions of three biosynthetic genes: two alanine racemase genes, *alr1* and *alr2* (SE1674 and SE1079), and the d-alanine aminotransferase gene, *dat* (SE1423). These three deletions restricted growth in d-alanine-deficient medium, pooled human blood, and skin. In the presence of d-alanine, SE_ΔΔΔ_ colonized and increased expression of human β-defensin 2 in cultured human skin models *in vitro.* SE_ΔΔΔ_ showed a low propensity to revert to d-alanine prototrophy and did not form biofilms on plastic *in vitro.* These studies support the potential safety and utility of SE_ΔΔΔ_ as a live biotherapeutic strain whose growth can be controlled by d-alanine.

**IMPORTANCE** The skin microbiome is rich in opportunities for novel therapeutics for skin diseases, and synthetic biology offers the advantage of providing novel functionality or therapeutic benefit to live biotherapeutic products. The development of novel bacterial strains whose growth can be controlled without the use of antibiotics or genetic elements conferring antibiotic resistance enables modulation of therapeutic exposure and improves safety. This study presents the design and *in vitro* evidence of a skin commensal whose growth can be controlled through d-alanine. The basis of this strain will support future clinical studies of this strain in humans.

## Abstract (precis)

The skin microbiome is rich in opportunities for novel therapeutics for skin diseases, and synthetic biology offers the advantage of providing novel functionality or therapeutic benefit to live biotherapeutic products. The development of novel bacterial strains whose growth can be controlled without the use of antibiotics or genetic elements conferring antibiotic resistance enables modulation of therapeutic exposure and improves safety. This study presents the design and *in vitro* evidence of a skin commensal whose growth can be controlled through d-alanine. The basis of this strain will support future clinical studies of this strain in humans.

## INTRODUCTION

Commensal microorganisms play a crucial role in maintaining human health across a number of organ systems, particularly in the skin (1–7). Diverse communities of microorganisms populate the skin, and a square centimeter can contain up to a billion microorganisms (8). These diverse communities of bacteria, fungi, mites, and viruses can provide protection against disease and form dynamic, yet distinct, niches on the skin (9). Increasing evidence has associated altered microbial communities or dysbiosis in the skin with cutaneous diseases (8, 10), especially atopic dermatitis (11–13). New strategies have emerged using microbes as therapy for treating a range of diseases (14). While this approach has gained particular attention in developing biotherapeutics for gastrointestinal diseases (15, 16), few studies have reported on using live microbes to treat skin diseases.

Staphylococcus epidermidis, recently dubbed the “microbial guardian of skin health” (17), is a strong candidate for use as a live biotherapeutic product (LBP) for skin conditions. S. epidermidis is a Gram-positive bacterium that is ubiquitous in the human skin and mucosal flora. As one of the earliest colonizers of the skin after birth, S. epidermidis plays an important role in cutaneous immunity and maintaining microbial community homeostasis (18, 19). In the clinical setting, S. epidermidis has demonstrated activity as a potential therapeutic (20–22). In Japan, in a double-blind, randomized clinical trial, topical application of autologous S. epidermidis in healthy volunteers increased lipid content of the skin, suppressed water evaporation, and improved skin moisture retention without signs of erythema (20). Other investigators have shown that S. epidermidis is capable of producing antimicrobial peptides (AMPs) that selectively target Staphylococcus aureus (23, 24). S. epidermidis has also shown other potent, therapeutic effects in preclinical settings. For example, a recent report described antineoplastic properties of certain strains of S. epidermidis (21). Naik et al. found that S. epidermidis (from the A20 clade) applied to the skin of germfree mice resulted in the development of protection against specific cutaneous pathogens (25). Other studies have shown that S. epidermidis may enhance innate skin immunity and limit pathogen invasion (19). In addition, S. epidermidis produces lipoteichoic acid, which can suppress Toll-like receptor 3 (TLR3)-mediated inflammation and protect mice from S. aureus infection (26, 27). Moreover, recent evidence suggests that topical S. epidermidis application is able to induce a nonclassical major histocompatibility complex class I (MHC-I)-restricted immune response to not only promote protection against pathogens but also accelerate wound repair in skin (18). The introduction of S. epidermidis could also aid in reestablishing skin homeostasis by targeting pathogen-driven dysbiosis.

Translational use of S. epidermidis in humans ideally requires controlling the viability and growth of the microbe, particularly in the manipulation, formulation, and life span of the therapeutic strain. While genetic manipulation of the strain using classical antibiotic selection markers could achieve this control, this method is discouraged by the Food and Drug Administration (FDA) for use in the final design of an LBP (14). Here, we propose an elegant solution for controlling an S. epidermidis strain for therapeutic use by introducing d-alanine auxotrophy. d-Alanine is required for the synthesis of peptidoglycans, which are essential components of the bacterial cell wall. d-Alanine is not normally present at significant levels in human tissue; so bacteria must produce it biosynthetically, or it must be supplied exogenously. In bacteria, biosynthesis is accomplished through the action of alanine racemase, which interconverts l-alanine and d-alanine, and by d-alanine aminotransferase, which interconverts d-glutamic acid and d-alanine. The metabolic pathway requirement for d-alanine auxotrophy varies in different genetic backgrounds. It was reported that the presence of glutamate racemase (interconverting d-glutamate and l-glutamate) with d-alanine aminotransferase (interconverting d-alanine and d-glutamate) provides a bypass for alanine racemase in S. aureus and Listeria monocytogenes (28, 29). In the present study, we found that, in S. epidermidis, it was necessary to knock out the d-alanine aminotransferase gene (SE1423) in addition to the two alanine racemase enzymes, *alr1* (SE1674) and *alr2* (SE1079), to fully confer the auxotrophic phenotype.

Here, we describe a strategy for generating a d-alanine-auxotrophic strain of S. epidermidis. This approach permitted tunable and precise control of growth of the organism. We further characterized its growth in culture and human blood and its colonization and activity in cultured skin models *in vitro.*

## RESULTS

**FIG 1:**
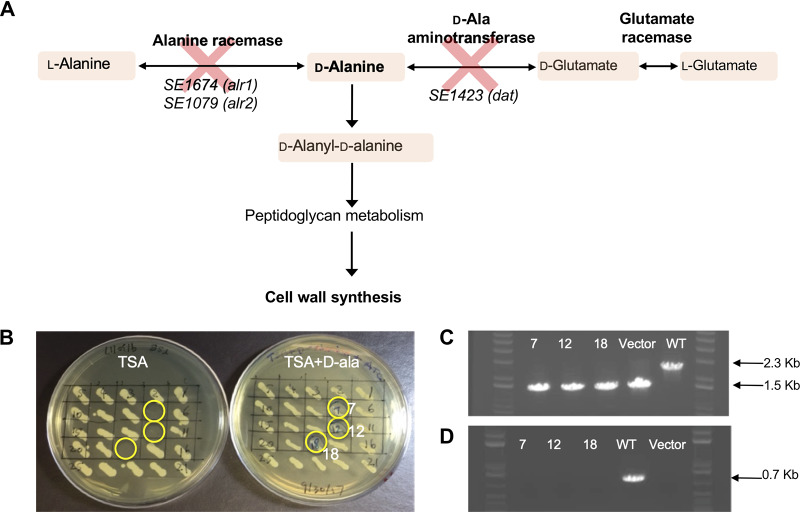
Strategy for d-alanine auxotrophy. (A) Alanine metabolism in staphylococcal species and the strategy for constructing a S. epidermidis d-alanine auxotroph. (B) The construction of S. epidermidis Δ*alr1* Δ*alr2* Δ*dat* is described in Materials and Methods. Twenty-five candidate clones were patched onto two different plates, and the plates were incubated at 30°C overnight. Left, TSA plate; right, TSA plus anhydrotetracycline (2 μg/ml) and d-alanine (40 μg/ml). Three clones (7, 12, and 18, highlighted in yellow circles) could only grow on TSA supplemented with d-alanine. (C and D) Example of PCR confirmation of gene knockout for S. epidermidis 1423 in SE_ΔΔΔ_ candidates. The DNA from cells patched onto a plate containing TSA plus anhydrotetracycline (2 μg/ml) and d-alanine (40 μg/ml) was used as the template in PCRs. Gel lanes are shown for the indicated knockout clones (7, 12, and 18), wild-type (WT) S. epidermidis, and SE1423KO plasmid DNA (Vector; as a control). For the experiment shown in panel C, PCR was performed using primers 1423-5F and 1423-3R to distinguish the wild-type SE1423 locus (PCR product of 2.3 kb) and the SE1423 knockout (PCR product of 1.5 kb). For the experiment shown in panel D, PCR was performed using primers 1423-F and 1423-R to detect a PCR product of 0.7 kb, specific for the wild-type SE1423 locus. As expected, the PCR products were not generated from the SE1423 knockout plasmid and putative SE1423 knockout S. epidermidis clones. Results confirmed successful SE1423 deletion in clones 7, 12, and 18, and similar PCR analysis was done to confirm deletions of SE1674 and SE1079.

Knocking out both alanine racemase genes, *alr1* and *alr2*, in S. epidermidis NRRL B-4268 (Δ*alr1* Δ*alr2*) did not produce an auxotrophic phenotype for d-alanine, in contrast to similar genetic knockouts in other bacteria such as Bacillus subtilis (30). This suggested that there was another metabolic pathway that could result in the production of d-alanine. The *dat* gene, encoding d-alanine aminotransferase, was identified as a candidate gene to knock out in order to eliminate potential interconversion of d-glutamate to d-alanine (Fig. 1A). The resulting triple-knockout strain, S. epidermidis Δ*alr1* Δ*alr2* Δ*dat* (SE_ΔΔΔ_), was successfully generated and displayed the desired d-alanine auxotrophy. All candidate clones (numbers 7, 12, and 18) showed some growth in an initial screen on tryptic soy agar (TSA) medium supplemented with 40 μg/ml d-alanine and failed to grow in the absence of supplementation (Fig. 1B). All three chromosomal deletions were similarly confirmed by PCR in each of the three candidate clones, numbers 7, 12, and 18, as illustrated in the example for SE1423 shown in Fig. 1C and D.

During strain construction, it was also noted that a Δ*dat* mutation alone in the absence of the Δ*alr1* Δ*alr2* mutations had no effect on the growth of the mutated strain in the absence of d-alanine (data not shown). Further, attempts to engineer a mutation in the glutamate racemase gene, *murI*, were unsuccessful, possibly due to the multifunctional nature of this protein, which has been observed in other bacteria (data not shown) (31, 32). The entire genomes of the S. epidermidis Δ*alr1* Δ*alr2* Δ*dat* mutant and parental strain NRRL B-4268, as well as the genome of the ATCC 12228 strain, were sequenced using a long-read methodology (PacBio, Menlo Park, CA) and assembled; no significant differences from the published sequences were observed except intended deletions in the *alr1*, *alr2*, and *dat* genes in the mutant (data not shown) (ATCC 12228; GenBank accession numbers AE015929 and CP043845). Further, complementation of the d-alanine auxotrophy by a plasmid containing the B. subtilis alanine racemase gene (*alrA*) confirmed that the auxotrophy was due to disruption of the d-alanine biosynthetic pathway, as expected from the three engineered deletions (data not shown).

In order to determine the frequency of reversion from d-alanine auxotrophy to prototrophy of SE_ΔΔΔ_, concentrated cell suspensions were plated on Vegitone agar without d-alanine supplementation and compared to cells on plates with d-alanine. The result indicates that no phenotypic revertants developed in the absence of d-alanine, with an estimated frequency of reversion of less than 4.8 × 10^−11^ revertants/CFU plated. In the same strain, the frequency of spontaneous rifampin resistance emergence was 6 × 10^−8^ mutants/CFU plated, demonstrating that the conditions of this experiment were able to select for mutations in strain SE_ΔΔΔ_ (see Fig. S1 in the supplemental material).

### Characteristics of SE_ΔΔΔ_ growth in broth cultures.

**FIG 2:**
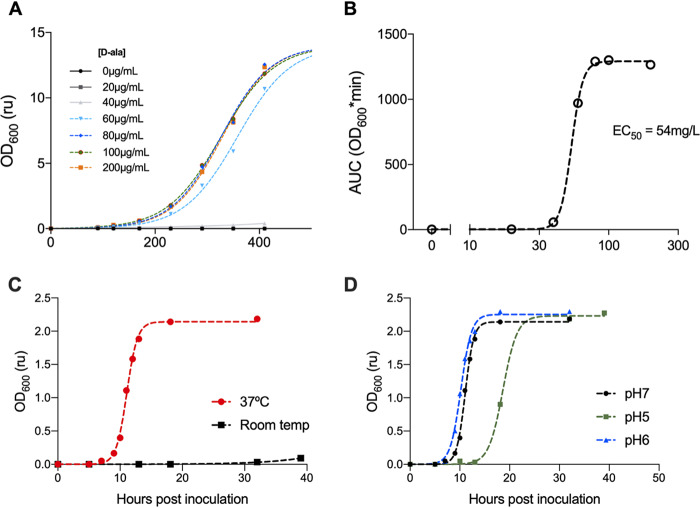
Characterization of SE_ΔΔΔ_ growth *in vitro.* (A) Graph showing the effect of d-alanine concentration (from 0 to 20 μg/ml) on the change in the OD at 600 nm over the time of incubation at 37°C for SE_ΔΔΔ_. Each data point is the mean ± standard deviation of three independent replicates. Dashed lines are the results from the logistic fit. As described in Materials and Methods, cultures were inoculated to a starting OD_600_ of 0.1, which corresponded to approximately 1 × 10^7^ CFU/ml. (B) EC_50_ curve of d-alanine concentration on the area under the concentration-time curve (AUC). (C) Graph showing the effect of temperature on the growth kinetics of SE_ΔΔΔ_. Growth curves were generated at room temperature and at 37°C using a d-alanine concentration of 100 μg/ml. One culture was placed into the incubator shaker (37°C; 250 rpm), and the second culture was placed at room temperature in a shaker (250 rpm). (D) Graph showing the effect of pH on the growth kinetics of SE_ΔΔΔ_. Growth curves, at 37°C, were generated from Vegitone medium that had been adjusted to pH 5 or 6 or the standard pH 7.0. d-Alanine was then added to obtain a final concentration of 100 μg/ml.

The kinetics of SE_ΔΔΔ_ growth in Vegitone broth were evaluated at 37°C with increasing concentrations of d-alanine (Fig. 2A). After ∼7 h, growth approached stationary phase, independently of the d-alanine concentration, such that generation times could be estimated using a standard logistic equation. From this, the estimated generation time was ∼31 to 45 min. No growth of the SE_ΔΔΔ_ strain was measured in the absence of d-alanine after 7 h, in agreement with the auxotrophic nature of the bacteria. There was no correlation between generation time and d-alanine concentration. The growth of SE_ΔΔΔ_ was very sensitive to the d-alanine concentration, as indicated by a steep Hill slope (∼11) of the concentration-growth curve (data not shown). The d-alanine concentration needed for the half-maximal response (50% effective concentration [EC_50_]) was 54 μg/ml (0.005%) (Fig. 2B). As there was no difference in the growth curves between conditions using 80 and 200 μg/ml of d-alanine, the standard concentration of d-alanine for routine growth of the auxotroph was set at 100 μg/ml. The growth of the auxotroph SE_ΔΔΔ_ strain in the presence of 100 μg/ml of d-alanine was similar to that of the wild-type parent, NRRL B-4268, at 37°C, further supporting that the only phenotype conferred by the triple deletion is d-alanine auxotrophy (Fig. S2).

10.1128/mSphere.00360-20.1 FIG S1 Frequency of spontaneous reversion of d-alanine auxotrophy in SE_ΔΔΔ_. Vegitone agar plates were inoculated with 2.1 ×10^10^ CFU of SE_ΔΔΔ._ and grown at 37 °C for 24 h without d-alanine (left), with 100 μg/ml d-alanine (middle), and in Vegitone agar supplemented with 100 μg/ml d-alanine and 8 ng/ml of rifampin (positive control) (right). A representative result of four independent trials is shown. The frequency of spontaneous resistance against rifampin observed here is consistent with literature reports for this antibiotic. Download FIG S1, PDF file, 0.5 MB. Copyright © 2020 Dodds et al. 2020 Dodds et al. This content is distributed under the terms of the Creative Commons Attribution 4.0 International license.

10.1128/mSphere.00360-20.2 FIG S2 A comparison of the growth of the d-alanine auxotroph SE_ΔΔΔ_ and the wild-type parental strain NRRL B-4268 in Vegitone medium. The growth of S. epidermidis SE_ΔΔΔ_ and NRRL B-4268 was evaluated at 37 °C in Vegitone broth as described in Materials and Methods, with the exception that cultures were inoculated from a suspension of an overnight agar plate to achieve a starting density of 10^5^ CFU/ml. d-Alanine (100 μg/ml) was added to the medium for SE_ΔΔΔ_. Growth was measured by the optical density at 600 nm and by quantifying CFU/ml by serial dilution. Representative results of replicate cultures are shown. Download FIG S2, PDF file, 0.2 MB. Copyright © 2020 Dodds et al. 2020 Dodds et al. This content is distributed under the terms of the Creative Commons Attribution 4.0 International license.

At room temperature, SE_ΔΔΔ_ showed little to no growth, as monitored by the optical density at 600 nm (OD_600_) (Fig. 2C) though the culture density increased by ∼2 log_10_ CFU over a 30-h time period (data not shown). The comparator culture grown at 37°C had a log_10_ CFU increase of ∼6 (data not shown) and reached stationary phase within ∼4 h from the end of lag phase (Fig. 2C).

Skin surface pH ranges from ∼4 to 6, compared to below the skin surface, which has a pH around 7 (34); therefore growth at lower pH in broth cultures was evaluated. SE_ΔΔΔ_ cultures grown at 37°C in standard Vegitone broth pH (7.0) and in broth adjusted to pH 6.0 each increased by ∼6 log_10_ CFU (data not shown) and reached stationary phase within 4 h from the end of the lag phase, showing generation times of 45.5 min and 35.5 min, respectively (Fig. 2D). The growth rate at pH 5.0 was somewhat lower (generation time of 65 min) and showed a more pronounced lag phase, yet cultures grew to about the same final cell densities as with medium at pH 6 and 7 (Fig. 2D). SE_ΔΔΔ_ showed no growth at pH 4 (data not shown).

### Characteristics of SE_ΔΔΔ_ safety.

We looked at three characteristics of SE_ΔΔΔ_ related to safety: (i) the ability of SE_ΔΔΔ_ to grow in human blood, (ii) its antibiotic resistance/susceptibility, and (iii) its potential to grow a biofilm. To test the ability of SE_ΔΔΔ_ to grow should it unintentionally enter the bloodstream, growth was assessed for 24 h in defibrinated human blood. SE_ΔΔΔ_ inoculated in human blood without d-alanine showed no growth after an overnight incubation with inocula of less than 1.4 × 10^8^ CFU/ml, but human blood supplemented with d-alanine was capable of supporting growth at all levels of inocula tested (except the lowest inoculum concentration of 1 × 10^0^) (Table S3). The CFU counts of blood cultures inoculated with 1.4 × 10^8^ CFU/ml remained constant, with no further growth or loss of viability under the conditions of this assay.

The antibiotic susceptibility profiles of NRRL B-4268 and SE_ΔΔΔ_ were determined using CLSI methodology. Table 1 shows that the SE_ΔΔΔ_ strain has antibiotic susceptibility similar to that of the parent wild-type S. epidermidis NRRL B-4268, and results were consistent with a prior literature report (35).

**TABLE 1:** Antibiotic susceptibility of SE_ΔΔΔ_ versus the parent S. epidermidis strain

Antibiotic	MIC (μg/ml)		Susceptibility^*a*^
	SE_ΔΔΔ_	NRRL B-4268	
Ampicillin	4	4	NA
Bacitracin	16	16	NA
Cefalexin	2	2	NA
Ceftaroline	0.12	0.12	S
Chloramphenicol	2	2	S
Clindamycin	0.06	0.06	S
Daptomycin	0.5	0.5	S
Doxycycline	4	4	S
Erythromycin	≤0.12	≤0.12	S
Fosfomycin	4	4	NA
Fusidic acid	≤0.03	≤0.03	NA
Gentamicin	≤0.06	≤0.06	S
Levofloxacin	0.12	0.12	S
Linezolid	≤0.5	≤0.5	S
Mupirocin	0.12	0.12	NA
Oritavancin	0.03	0.06	S
Oxacillin	0.12	0.12	S
Quinupristin/dalfopristin	≤0.12	≤0.12	S
Rifampin	≤0.002	≤0.002	S
Tetracycline	>32	>32	R
Tigecycline	0.25	0.12 - 0.25	S
Trimethoprim/sulfamethoxazole	≤0.5/9.5	≤0.5/9.5	S
Vancomycin	1	1	S

a According to CLSI breakpoints: S, sensitive; R, resistant. NA, not available.

**FIG 3:**
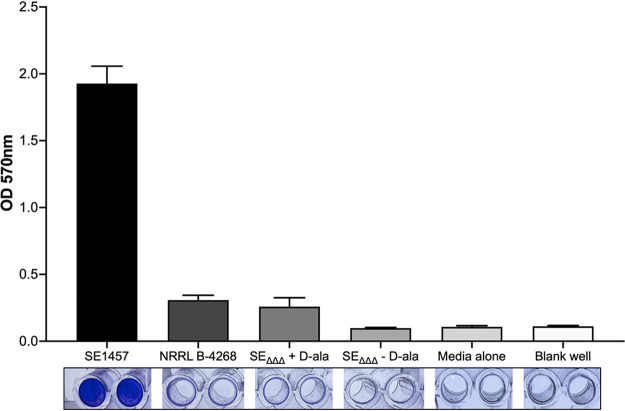
The production of biofilm from S. epidermidis *in vitro*. The bottom panel shows the accumulation of biofilm on plastic wells within 24 h at 37°C, visualized by crystal violet staining. Biofilm formation was further quantified by dissolving the crystal violet in acetic acid and reading absorbance at 570 nm. S. epidermidis strain 1457 was the positive control, and the blank and TSB medium-only wells served as negative controls for background staining. All data shown are means ± standard deviations of 16 replicate microtiter wells.

Finally, we looked at the ability of SE_ΔΔΔ_ to form biofilm. This experiment was performed on polystyrene plastic, and growth was evaluated in a standard crystal violet assay in a 96-well format. Cultures were grown in tryptic soy broth (TSB)–0.5% d-glucose, with and without d-alanine, and biofilm formation was detected and quantified using crystal violet. The data in Fig. 3 show that the crystal violet staining for SE_ΔΔΔ_, with or without d-alanine present, was indistinguishable from that of the blank and TSB controls, indicating that no biofilms formed on the plastic under the conditions of this assay. Wells containing the positive-control strain, SE1457, showed strong crystal violet staining, consistent with this strain’s known *ica-*positive genotype and biofilm-forming phenotype.

### SE_ΔΔΔ_ colonization of a human skin model *in vitro*.

**FIG 4:**
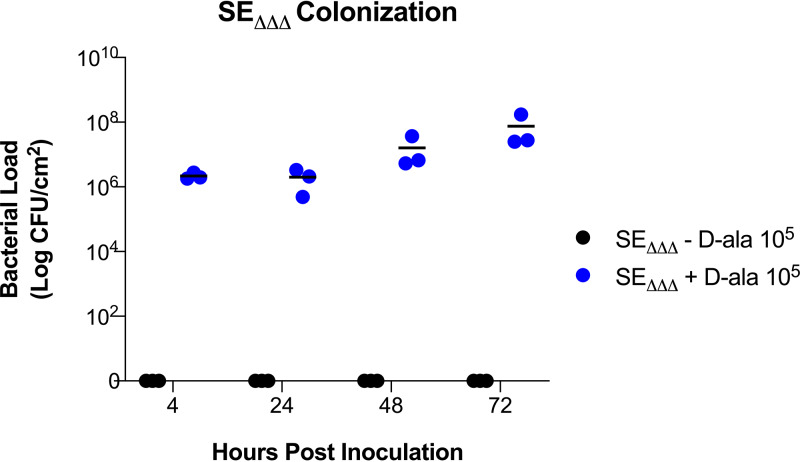
SE_ΔΔΔ_ growth in RHE. RHE inserts were colonized with SE_ΔΔΔ_ at 37°C in 5% CO_2_. d-Alanine at 100 μg/ml (+ d-Ala) was added to the medium feeding the RHE insert, as indicated. Colonized models were harvested at each time point by uniform skin punches. Skin punch samples were rinsed in DPBS to remove nonadherent cells, vortexed, and assayed for cell density by serial dilution and plating on SaSelect plates supplemented with 100 μg/ml of d-alanine. Each condition was tested in triplicate.

To determine if d-alanine supplementation is necessary for SE_ΔΔΔ_ colonization and survival on reconstructed human epidermis (RHE), 10^5^ CFU of SE_ΔΔΔ_ was inoculated onto the RHE surface either with or without d-alanine supplementation. No colonization occurred without d-alanine present, indicating that d-alanine must be supplied for growth on skin (Fig. 4). Furthermore, these data show that SE_ΔΔΔ_ colonized the RHE within 4 h and persisted for up to 72 h following a single application with d-alanine supplementation. Interestingly, as early as 4 h postinoculation, in the absence of d-alanine, no bacteria were recovered from the RHE when homogenates were plated on SaSelect plates supplemented with d-alanine.

**FIG 5:**
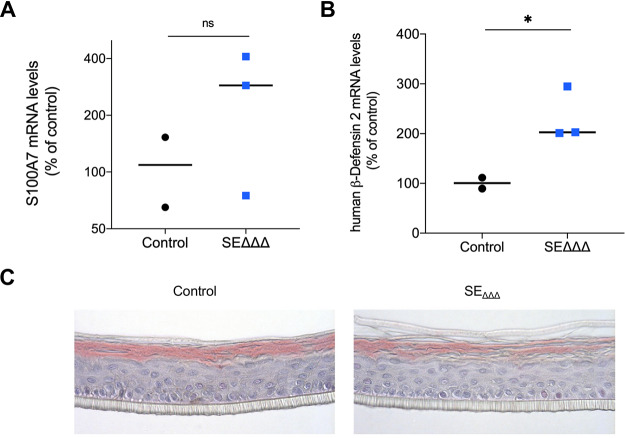
Expression of human AMPs following SE_ΔΔΔ_ treatment of RHE. (A and B) Plots of the changes in mRNA levels for two epithelial AMPs (S100A7 and hβD-2). hβD-2 was elevated in the presence of SE_ΔΔΔ_. (C) Hematoxylin and eosin staining of transverse sections of RHE inserts with or without treatment with SE_ΔΔΔ_. The vehicle control was PBS containing 100 μg/ml d-alanine. ns, not significant. *, *P* < 0.05. Horizontal lines indicate medians.

SE_ΔΔΔ_ was also evaluated for its ability to trigger host-commensal communication. SE_ΔΔΔ_ was seeded on RHE inserts, and mRNA levels for specific host-commensal signals were measured by quantitative PCR (qPCR). In Fig. 5, data show a trend in the change in expression levels of two epithelial AMPs, S100 calcium-binding protein A7 (S100A7) and human β-defensin 2 (hβD-2). In this sample (*n* = 3), the average expression levels of both AMPs were heightened in the presence of SE_ΔΔΔ_. No deleterious effects of the SE_ΔΔΔ_ colonization on the structure of the RHE were observed histologically.

## DISCUSSION

A strategy is presented for the construction of an S. epidermidis strain, SE_ΔΔΔ_, with a non-antibiotic-associated conditional growth phenotype. SE_ΔΔΔ,_ exhibits key features that support its potential as a skin LBP: its growth is tunable with d-alanine, its d-alanine auxotrophy has a low propensity to phenotypically revert, it colonizes human skin equivalents, it does not grow in pooled human blood, it does not form biofilms on plastic, and it increases expression of an important host AMP, hβD-2. Our strategy involved deleting the ability to synthesize d-alanine, which is required for the synthesis of bacterial cell peptidoglycan. Here, we show that, as expected, disruption of peptidoglycan through d-alanine starvation is bactericidal in culture and on skin *in vitro*. In contrast to reports for B. subtilis, in order to develop d-alanine auxotrophy in S. epidermidis, not only the two alanine racemase genes (*alr1* and *alr2*) but also the d-alanine aminotransferase gene, *dat*, had to be knocked out. Evidently, the combination of glutamate racemase and d-alanine aminotransferase provides a viable bypass to alanine racemase, as reported in MRSA132 (28) and Listeria monocytogenes (29). Other investigators have reported on other auxotrophy strategies for attenuating growth in S. aureus, including proline (36), menadione (37), and NK41 (38).

Several findings from these studies support the idea that attenuation by d-alanine auxotrophy may promote the safety of SE_ΔΔΔ_ for use as a skin LBP. The very low spontaneous reversion rate of d-alanine auxotrophy suggests that very large doses of SE_ΔΔΔ_ may be applied to skin without selecting for a prototrophic revertant. Further, results also support that the d-alanine auxotrophy of SE_ΔΔΔ_ may mitigate the risk of bacterial dissemination into the bloodstream. Biofilm-forming S. epidermidis is often associated with opportunistic nosocomial infections due to the ability to colonize biomaterials of indwelling medical devices (39, 40). Therefore, biofilm formation is an undesirable property of an LBP. No biofilm formation was seen with SE_ΔΔΔ_, with and without d-alanine, suggesting that this strain may have a lower propensity to form biofilm in a clinical setting. Another important observation is that the inability to form biofilm *in vitro* did not affect the ability of SE_ΔΔΔ_ to robustly colonize and grow on RHE.

S. epidermidis belongs to the group of coagulase-negative staphylococci (CoNS), which is distinguished from coagulase-positive staphylococci such as S. aureus by lacking the enzyme coagulase. A large arsenal of tools is available to S. epidermidis to control the colonization of other pathogenic microorganisms, including proteases that degrade proteins in S. aureus biofilm, stimulation of AMP secretion from keratinocytes, or secretion of lantibiotics targeting S. aureus (41–47). In addition, S. epidermidis produces lipoteichoic acid, which can suppress TLR3-mediated inflammation and protect mice from S. aureus infection (48). Specific novel lantibiotics secreted by S. epidermidis and Staphylococcus hominis were recently shown to control S. aureus infection by synergizing with the human keratinocyte-secreting cathelicidin-related AMP LL-37 (49). This is a further demonstration of the important and beneficial interplay between commensals and the host. Interestingly, these lantibiotic-secreting strains were significantly underrepresented in the microbiome of atopic dermatitis patients, and exogenous application of these lantibiotic-secreting commensals resulted in a reduction in S. aureus infection in a small group of atopic dermatitis patients (49). Introduction of d-alanine auxotrophy into other S. epidermidis strain backgrounds may be a way of broadly controlling isolates selected for their novel therapeutic properties.

Skin innate immunity is the first line of defense after the physical skin barrier. Keratinocytes are the main skin cell population involved (50–52); they express a variety of TLRs, which are primary sensors of innate immunity (53, 54). The innate immune system can recognize pathogens and trigger the host response to eliminate them via the release of cytokines (e.g., interleukin-1α [IL-1α] and IL-1β) and AMPs (51, 55–57). AMPs are secreted by keratinocytes, sebocytes, T cells, and mast cells and can directly attack pathogens (e.g., bacteria, enveloped viruses, and fungi) (58–61). AMPs can be grouped into five separate classes: defensins, dermcidin, cathelicidins, S100 proteins, and ribonucleases (61–63). Some are constitutively expressed while others can be induced following a skin insult. AMPs are highly cationic and thus interact with negatively charged membrane components (e.g., lipopolysaccharide [LPS], peptidoglycans, outer membrane protein I [OprI]) of skin pathogens, essentially forming holes in the cell membrane (58). AMPs have also been shown to exert immunomodulatory effects, interacting directly with receptors on immune cells to promote chemotaxis, differentiation, and cytokine production (39). The most abundant AMPs in human skin are human defensins, the cathelicidin LL-37, and RNase 7. Several studies have shown that commensal organisms such as S. epidermidis can directly induce expression of hβD-2 (*hDEFB4A* gene) while presenting a relative tolerance to it, enabling such organisms to survive on the skin surface and to modulate defensin expression when the stratum corneum barrier is disrupted (64). Therefore, commensal bacteria such as S. epidermidis are able to amplify the innate immune response of human keratinocytes to pathogens by induction of AMP expression (62). Our SE_ΔΔΔ_ auxotroph promoted the expression hβD-2, a key AMP in the skin that has been shown to inhibit S. aureus, thus demonstrating its ability to favorably modulate host innate immunity (65).

This study had several limitations, including the restriction of using reconstructed human epidermis instead of *in vivo* studies. However, given evidence that S. epidermidis does not natively colonize mice and the poor translation of microbiome studies in mice to humans (66), *in vivo* studies themselves carry limitations. Three-dimensional *in vitro* skin models like the RHE we developed in this study have been established to study human microbiota on the skin as an alternative to *in vivo* studies (67). Future clinical testing of SE_ΔΔΔ_ in humans will provide key information *in vivo* such as growth on live skin, competition with other microbes, and safety.

In this study, we describe the engineering of a live skin commensal, S. epidermidis, and characterize its favorable properties which support its potential use as an LBP for skin diseases. Properties conferred by the d-alanine auxotrophy and the strain background itself support the potential of SE_ΔΔΔ_ to serve either as an LBP itself or as a recombinant host for expressing novel skin therapeutics. Future studies will evaluate the effect of this LBP candidate on human skin in the clinical setting.

## MATERIALS AND METHODS

### Generation and confirmation of auxotrophy.

The commensal, nonpathogenic strain S. epidermidis, NRRL B-4268 was obtained from the USDA (Agricultural Research Service, NRRL culture collection, Peoria, IL, USA). This strain is also known as ATCC 12228 (American Type Culture Collection, Manassas, VA, USA) and PCI 1200 (U.S. FDA). This strain was selected for its low virulence potential because it lacks the *ica* operon implicated in S. epidermidis*-*associated catheter bloodstream infections (68).

The reference genome has been published as the ATCC 12228 genome (35, 69). The genome encodes two annotated alanine racemase genes, SE1674 (*alr1*) and SE1079 (*alr2*); both genes were targeted for deletion. The strategy for conferring d-alanine auxotrophy to NRRL B-4268 is presented in Fig. 1A Briefly, chromosomal DNA from S. epidermidis NRRL B-4268 was isolated and used as a template for PCR. The 5′ flanking region (1.0 kb) of SE1674 was amplified using forward primer 1674-5F (SalI) and reverse primer 1674-5R. Similarly, the 3′ flanking region (1.0 kb) of SE1674 was amplified using forward primer 1674-3F and reverse primer 1674-3R (EcoRI). Overlapping PCR was performed using a mixture of the 5′ PCR and 3′ PCR products as templates, and primers 1674-5F (SalI) and 1674-3R (EcoRI) to generate a PCR product, termed 5′-1674-3′ (2.0 kb).

The overlapping PCR product 5′-1674-3′ was digested with SalI and EcoRI, cloned into the temperature-sensitive plasmid pJB38 at the EcoRI-SalI sites (70), and transformed into TOP10 Escherichia coli (Life Technologies, Inc., Carlsbad, CA, USA) using ampicillin selection (100 μg/ml) to develop knockout (KO) plasmid pJB-1674KO that contains a chloramphenicol (Cm) resistance selection marker. Plasmid pJB-1674KO was purified and transformed into E. coli GM2163 (a *dam dcm* mutant) using ampicillin selection (100 μg/ml) and then purified and transformed into NRRL B-4268 by electroporation. Transformants were grown at the permissive temperature (30°C) on tryptic soy agar plates (TSA) containing 10 μg/ml Cm. (see Tables S1 and S2 in the supplemental material for more information on the strains, plasmids, and primers used.)

10.1128/mSphere.00360-20.3 TABLE S1 Strains and plasmids used or developed in this study. Download Table S1, DOCX file, 0.02 MB. Copyright © 2020 Dodds et al. 2020 Dodds et al. This content is distributed under the terms of the Creative Commons Attribution 4.0 International license.

10.1128/mSphere.00360-20.4 TABLE S2 Primers used in this study. Download Table S2, DOCX file, 0.01 MB. Copyright © 2020 Dodds et al. 2020 Dodds et al. This content is distributed under the terms of the Creative Commons Attribution 4.0 International license.

10.1128/mSphere.00360-20.5 TABLE S3 Growth and survival of SE_ΔΔΔ_ in defibrinated pooled healthy human blood after 24 h of incubation. Download Table S3, DOCX file, 0.01 MB. Copyright © 2020 Dodds et al. 2020 Dodds et al. This content is distributed under the terms of the Creative Commons Attribution 4.0 International license.

For each of the gene knockouts created in this study, the presence of each pJB38 KO plasmid in initial S. epidermidis transformants was confirmed by PCR amplification of the flanking region of each gene. Colonies of confirmed pJB38 KO plasmid transformants were restreaked onto fresh TSA with Cm (10 μg/ml) and d-alanine (40 μg/ml), and plates were incubated at 43°C for 24 h to select for plasmid integration into the chromosome via single-crossover homologous recombination. Isolated colonies were streaked again for purification at 43°C and then inoculated into 50 ml of tryptic soy broth (TSB) plus d-alanine (40 μg/ml), without Cm, in a 250-ml baffled shake flask and grown at 30°C for 24 h to permit the excision of plasmid sequences from the chromosome by homologous recombination. The culture was passaged six times by transferring an aliquot of 0.5 ml to a flask containing 50 ml of fresh medium and growing the culture at 30°C for 24 h. The final culture was plated on TSA with 2 μg/ml anhydrotetracycline (to induce plasmid counterselection) and d-alanine (40 μg/ml). After 2 days of incubation at 30°C, approximately 100 to 200 colonies were formed on plates inoculated with 100 μl of culture at a 10^−5^ dilution. Following integration of the KO plasmid and removal of the plasmid backbone containing the antibiotic selection marker, an SE1674 knockout strain (S. epidermidis Δ*alr1*) was isolated and confirmed by PCR amplification using primers flanking the gene. Using the same gene knockout method described above, we proceeded to knock out SE1079 in this strain, leading to successful generation of double-knockout strains (S. epidermidis Δ*alr1* Δ*alr2*). More information about the plasmids and PCR primers used in this study can be found in Tables S1 and S2, respectively.

### Measurement of effect of d-alanine levels on SE_ΔΔΔ_ growth.

The effect of d-alanine concentrations ranging from 0 μg/ml to 200 μg/ml (0% to 0.02%) on the short-term growth curves of SE_ΔΔΔ_ was investigated. Five milliliters of Vegitone medium (catalogue no. 41960; Sigma-Aldrich) containing 0.004% (40 μg/ml) d-alanine (99+%; Acros Organics) was inoculated with an isolated SE_ΔΔΔ_ colony. Cultures were incubated overnight at 37°C with shaking at 250 rpm on an orbital shaker.

Growth rates were measured at 37°C for up to 7 h. Fifty milliliters of Vegitone containing the prescribed amount of d-alanine was inoculated with 1 ml of the overnight culture to give a final optical density (OD) at 600 nm of 0.1 relative units (RU). Flasks were incubated on an orbital shaker at 250 rpm. Every ∼60 min, a sample was taken, and the OD_600_ was recorded. Growth curves were conducted for 7 h and were fitted using a standard logistic growth equation (71).

### Measurement of effect of temperature and pH on SE_ΔΔΔ_ growth.

The effect of temperature and medium pH on SE_ΔΔΔ_ growth were examined using 100 μg/ml d-alanine in all groups. Growth curves were measured at room temperature (∼25°C) and at 37°C for up to 40 h. Fifty milliliters of Vegitone containing d-alanine was inoculated with 1.0 ml of the overnight culture. Growth curves were also evaluated at three different pH values (pHs 4, 5, 6, and 7) at 37°C for 40 h.

### Antibiotic susceptibility testing of S. epidermidis NRRL B-4268 and SE_ΔΔΔ_.

Antibiotic susceptibility testing was conducted at IHMA Europe Sàrl, Monthey, Switzerland, using Clinical and Laboratory Standards Institute (CLSI) methodology, with the exception that 100 mg/liter d-alanine was added to testing with SE_ΔΔΔ_ (72).

### Determination of the frequency of spontaneous phenotypic reversion of auxotrophy of SE_ΔΔΔ_.

The d-alanine-deficient medium used to isolate revertants was Vegitone agar plates, prepared in 150-mm petri dishes. Rifampin stocks were prepared in methanol at a final concentration of 4 μg/ml. As appropriate, Vegitone agar plates were prepared with either 100 μg/ml of d-alanine or 8 ng/ml of rifampin and 100 μg/ml of d-alanine. Eight milliliters of the overnight culture was spun down at 10,000 rpm for 10 min. The pellets were resuspended in 1,200 μl of Vegitone broth, and 150 μl of the suspension was spread onto 150-mm petri dishes. All plates were incubated at 37°C for ∼24 h. The experiment was carried out in quadruplicate. The remaining inoculum suspension was serially diluted 1:10 in Vegitone broth and plated onto Vegitone agar plates supplemented with 100 μg/ml d-alanine and incubated at 37°C for 24 h to quantify CFU in the inoculum. The spontaneous resistance frequency was determined by dividing the number of colonies grown in in the absence of d-alanine by the total number of CFU plated.

### Determination of growth of SE_ΔΔΔ_ in blood.

TSB supplemented with 100 μg/ml of d-alanine was inoculated with SE_ΔΔΔ_ and incubated overnight at 37°C. The overnight culture was further diluted in fresh TSB plus d-alanine and allowed to grow until it reached mid-log phase, which corresponded to an absorbance of ∼0.7, as measured by the OD_600_. Virus-free, defibrinated pooled whole human blood from three healthy donors was purchased from BioIVT (Westbury, NY). The SE_ΔΔΔ_ culture was concentrated to 1 × 10^9^ CFU/ml. Serial 10-fold dilutions ranging from 1 × 10^9^ to 1 × 10^1^ were made in TSB with or without d-alanine and then diluted again 10-fold into a volume of 100 μl of blood in a 96-well microtiter plate. All blood cultures were incubated in a tissue culture incubator at 37°C in 5% CO_2_ for 24 h. The following day, cultures were serially diluted and plated on TSA with 100 μg/ml of d-alanine, grown overnight, and scored for the number of colonies. The experiment was carried out in duplicate. The remaining inoculum suspension was serially diluted in TSB and plated onto TSA supplemented with 100 μg/ml of d-alanine to quantify CFU in the inoculum. All plates were incubated at 37°C for 24 h.

### Growth of SE_ΔΔΔ_ on reconstructed human epidermis.

Fully differentiated antibiotic-free reconstructed human epidermis (RHE) culture inserts from Mattek (EpiDerm, Ashland, MA, USA) were used to evaluate bacterial colonization. RHE models were reactivated by placing cell inserts into six-well plates containing 1 ml of EpiDerm maintenance medium from Mattek. The inserts were then incubated for 18 h at 37°C in 5% CO_2_. Following incubation, the inserts were transferred to 12-well hanging culture inserts with 5 ml of Epiderm maintenance medium in each well, and the tissues were incubated for an additional 30 min at 37°C in 5% CO_2_. When applicable, 100 μg/ml of d-alanine was also added to the RHE medium. SE_ΔΔΔ_ was inoculated into 25 ml of TSB containing 100 μg/ml of d-alanine. The culture was grown at 37°C overnight with shaking. After 18 h, fresh TSB supplemented with 100 μg/ml of d-alanine was inoculated with the overnight culture of SE_ΔΔΔ_ at a starting OD_600_ of 0.1. Cultures were grown at 37°C to log phase (OD_600_ of 0.5 to 0.7), pelleted by centrifugation at 15,000 rpm for 2 min, and then resuspended in Dulbecco’s phosphate-buffered saline (DPBS) to a final OD_600_ of 1.0. The bacterial pellet was suspended in 1 ml of DPBS and pelleted at 15,000 rpm for 2 min. This process was repeated two more times to ensure that residual d-alanine was removed from the bacterial suspension. The bacterial cells were next diluted down to 0.01 OD_600_ in DPBS. For cells receiving d-alanine, 100 μg/ml of d-alanine was added to the bacterial suspension prior to RHE inoculation. To inoculate the RHE inserts, 10 μl of the prepared bacterial suspension was applied to the center of the dry surface-air interface of the RHE, ensuring that the liquid droplet remained centered when possible. The RHE inserts were then incubated at 37°C in 5% CO_2_ for 4 h, 24 h, 48 h, or 72 h. At each time point, bacterial colonization was assessed by taking a 4-mm punch biopsy specimen from the center of the RHE. Biopsy specimens were washed by submerging the tissue in 1 ml of DPBS to remove unadhered cells. In order to release the colonized bacterial cells from the RHE, the biopsy specimens were transferred to 1 ml of DPBS, followed by vortexing for 2 min at full speed. Colonization of SE_ΔΔΔ_ was quantified by dilution plating and counting CFU on SaSelect plates supplemented with 100 μg/ml d-alanine (Bio-Rad, Hercules, CA). All experiments were done in triplicate.

### Biofilm formation assay with SE_ΔΔΔ_.

The biofilm assay was adapted from Mack et al. and Neopane et al. (73, 74). TSB was inoculated with S. epidermidis strain SE_ΔΔΔ_ and the biofilm-forming strain S. epidermidis 1457 (75) and incubated overnight at 37°C. The culture was then diluted 1:200 in fresh TSB–0.5% glucose, and 200 μl was dispensed into a flat-bottom, 96-well tissue culture plate (Thermo Fisher Scientific, Waltham, MA). The plate was incubated at 37°C for 24 h. After incubation, the microtiter plate was washed twice with 1× DPBS to remove loosely attached bacterial and planktonic cells. The biofilms were fixed by incubation in 200 μl of 99% methanol for 10 min. The methanol was decanted, and wells were allowed to air dry for 10 min. Once dry, wells were stained with 0.1% crystal violet for 5 min. Excess crystal violet was washed twice with deionized water. Wells were air dried, and then stained biofilms were removed by dissolving them in 200 μl of 30% glacial acetic acid. The amount of biofilm was quantified by measuring absorbance of the plate at 570 nm.

### Effect of SE_ΔΔΔ_ on AMP production in cultured human skin.

The effect of SE_ΔΔΔ_ on antimicrobial peptide (AMP) production in cultured human skin was determined at StratiCELL (Les Isnes, Belgium) using RHE inserts. RHE inserts were cultured in triplicates at the air-liquid interface for 14 days in gentamicin-free culture medium containing d-alanine (100 μg/ml) in 95% humidity at 37°C with 5% CO_2_ to fully differentiate the RHE. A single topical treatment with SE_ΔΔΔ_ (10 μl at a cell density of 1 × 10^7^ CFU/ml in phosphate-buffered saline [PBS] plus 100 μg/ml d-alanine) was allowed to colonize for 48 h on fully differentiated epidermis. Control samples were treated with 10 μl of the vehicle solution (PBS plus d-alanine 100 μg/ml). During the treatment, the tissue culture medium feeding the RHE was also supplemented with 100 μg/ml d-alanine. RHE inserts were kept at the air-liquid interface in a humid atmosphere at 37°C with 5% CO_2_.

### Histological analysis of RHE.

At the end of the treatment, three tissue samples per condition were fixed in 4% formaldehyde, dehydrated, and paraffin embedded. Sections of 6 μm of epidermis were stained with eosin and hematoxylin. Slides were mounted with specific medium and examined with a Leica DM2000 photomicroscope coupled to a digital camera (Zeiss).

### Analysis of AMP expression in RHE.

To quantify gene expression of human AMPs, total mRNA was extracted using a Qiagen RNeasy kit (Hilden, Germany). Tissues were washed in PBS, removed from their inserts, and immersed directly in the lysis buffer. Extraction of mRNA was performed according to the supplier’s recommendations. The collected mRNA was stored at –80°C. Reverse transcription was performed with a high-capacity mRNA-to-cDNA kit (Applied Biosystems, Foster City, CA) from 1 μg of total mRNA, according to the manufacturer’s instructions.

The target sequences of the genes of interest, S100 calcium-binding protein A7 (S100A7) and human β-defensin 2 (DEFB4A), were amplified by using TaqMan gene expression assays (Applied Biosystems, Foster City, CA). TaqMan probes were grafted with a fluorophore (6-carboxyfluorescein [FAM]) at their 5′ ends and with a fluorescence quencher in the 3′ ends. PCRs were performed with a QuantStudio 7 real-time PCR system (Applied Biosystems, Foster City, CA). In order to normalize the results, a housekeeping gene (β2-microglobulin [B2M]) was used. The thermal cycles were programmed with first one denaturation step at 95°C for 20 s. The amplification protocol followed with 40 cycles (1s at 95°C and 20 s at 60°C). Threshold cycle (*C_T_*) values were obtained for each gene. Data were analyzed by using the relative quantification (RQ) application available on the Applied Biosystems website (and designed to perform relative quantification of gene expression using the comparative *C_T_* [ΔΔ*C_T_*] method) (76, 77).
