# Supplementary material for: Controlling the Growth of the Skin Commensal Staphylococcus epidermidis Using d-Alanine Auxotrophy
Source: mSphere. 2020 Jun 10;5(3):e00360-20. doi: 10.1128/mSphere.00360-20 (PMC7289707; doi:10.1128/mSphere.00360-20)

Condition:	No D-alanine	100 µg/mL D-alanine	8 ng/mL rifampicin/ 100 µg/mL D-alanine
Frequency:	**<4.8 x 10^{-11}**	**Confluent Growth**	**6 x 10^{-8}**

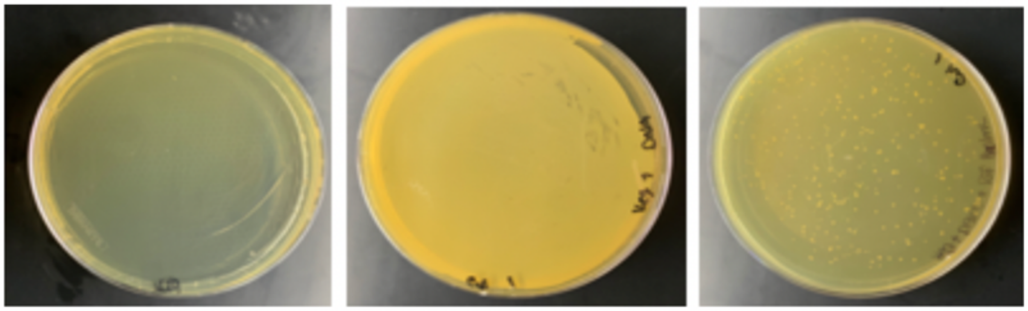

Supplement: FIG S1 [file mSphere.00360-20-sf001.pdf]
