# Supplementary figures and images for: Controlling the Growth of the Skin Commensal Staphylococcus epidermidis Using d-Alanine Auxotrophy
Source: mSphere. 2020 Jun 10;5(3):e00360-20. doi: 10.1128/mSphere.00360-20 (PMC7289707; doi:10.1128/mSphere.00360-20)

**A.**

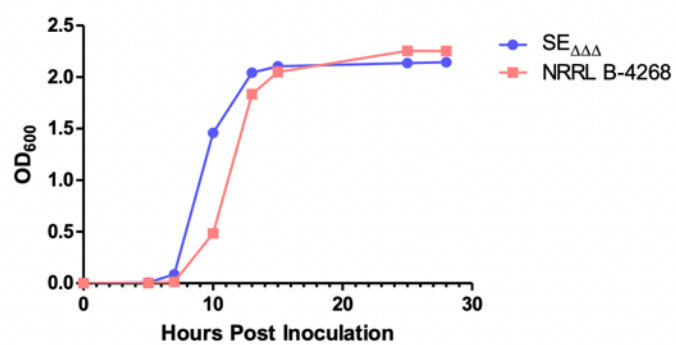

**B.**

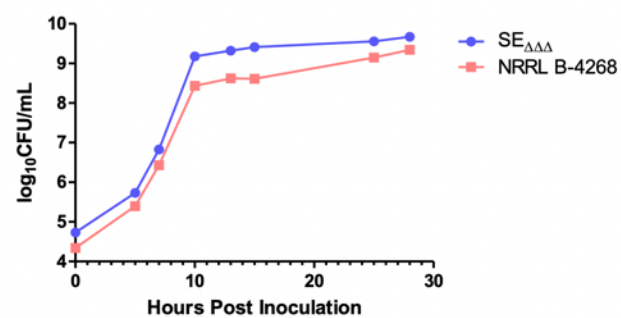

Supplement: FIG S2 [file mSphere.00360-20-sf002.pdf]
