# Supplementary material for: Controlling the Growth of the Skin Commensal Staphylococcus epidermidis Using d-Alanine Auxotrophy
Source: mSphere. 2020 Jun 10;5(3):e00360-20. doi: 10.1128/mSphere.00360-20 (PMC7289707; doi:10.1128/mSphere.00360-20)
Supplement: TABLE S1 [file mSphere.00360-20-st001.docx]

**Supplementary Table S1. Strains and plasmids used or developed in this study**

| **Strain or plasmid** | **Relevant characteristics** | **Source or reference** |
| --- | --- | --- |
| *Escherichia coli* | | |
| Top10 |  | Life Technologies, Inc., Carlsbad, CA, USA |
| GM2163 (*dam^-^/dcm^-^)* | Adenine and cytosine methylation-deficient *E. coli* | Source?New England Biolabs, Ipswich, MA, USA |
| *Staphylococcus epidermidis* |  |  |
| NRRL B-4268 | Non-biofilm-forming S. epidermidis strain. Originally deposited as PCI 1200. Re-deposited as ATCC 12228, then re-deposited as NRRL B-4268. | USDA ARS NRRL Collection  (Zhang, Ren et al. 2003) |
| SEΔ*alr1*Δ*alr2* | Alanine racemase-deficient NRRL B-4268 | This study |
| SEΔ*alr1*Δ*alr2*Δ*dat*, or SE_ΔΔΔ_ | d-alanine auxotrophic NRRL B-4268 | This study |
| 1457 | *Ica -* positive, biofilm-forming | (Galac, Stam et al. 2017) |
| *Staphylococcus aureus* |  |  |
| RN4220 | Restriction-deficient 8325-4 | (Kreiswirth, Lofdahl et al. 1983) |
| Plasmids |  |  |
| pJB38 | Allelic exchange shuttle vector, Cam^r^/ Amp^r^ | (Cheung, Bayer et al. 2004) |
| pJB38-1674KO | pJB38 containing the 5’ upstream and 3’ downstream flanking sequences of the target gene SE1674 | This study |
| pJB38-1079KO | pJB38 containing the 5’ upstream and 3’ downstream flanking sequences of SE1079 | This study |
| pJB38-1423KO | pJB38 containing the 5’ upstream and 3’ downstream flanking sequences of SE1423 | This study |
