# Supplementary material for: Controlling the Growth of the Skin Commensal Staphylococcus epidermidis Using d-Alanine Auxotrophy
Source: mSphere. 2020 Jun 10;5(3):e00360-20. doi: 10.1128/mSphere.00360-20 (PMC7289707; doi:10.1128/mSphere.00360-20)
Supplement: TABLE S2 [file mSphere.00360-20-st002.docx]

**Supplementary Table S2. Primers Used in This Study**

| **Name** | **Sequence (5’ to 3’)** | **Application** |
| --- | --- | --- |
| 1674-5F (SalI) | atgc**gtcgac**TTGGTACATGAAAGGTGATAC | To amplify the 5’ flanking region of SE1674 (1.0 Kb) |
| 1674-5R | caaatttcctaatcagtgactataATATATGTCCTCCTTGAAACTACTTAC |  |
| 1674-3F | gtaagtagtttcaaggaggacatatatTATAGTCACTGATTAGGAAATTTG | To amplify the 3’ flanking region of SE1674 (1.0 Kb) |
| 1674-3R (EcoRI) | acgt**gaattc**TTCCACGAAATGCGCCTC |  |
| 1674-F | ATGTCAGAGAAGTTTTATAGAG | To amplify the entire SE1674 CDS (1.2 Kb, deleted in 1674 KO strains) |
| 1674-R | CTATTTTAACAATTCGTTAGTAAC |  |
| JB-Cm-F | TTGATTTAGACAATTGGAAGAG | To amplify part of the chloramphenicol selection marker (0.7 Kb) in pJB38 |
| JB-Cm-R | AAGTACAGTCGGCATTATCTC |  |
| 1079-5F (EcoRI) | acgt**gaattc**GTTACATTGCACAGAAG | To amplify 5’ flanking region of SE1079 (1.2 Kb) |
| 1079-5R | ctccttcataagagaatcgtgTTGCTTTACACCTCTTTATAATTTC |  |
| 1079-3F | gaaattataaagaggtgtaaagcaaCACGATTCTCTTATGAAGGAG | To amplify 3’ flanking region of SE1079 (1.0 Kb) |
| 1079-3R (SalI) | acgt**gtcgac**ACGCCTCATACTGTGCACCATAAAG |  |
| 1079-F | TTGACAGCAATTTGGTCATTAG | To amplify SE1079 CDS (1.1 Kb, deleted in 1079 KO strains) |
| 1079-R | CTCCTTCATAAGAGAATCGTG |  |
| 1423-5F  (EcoRI) | atgc**gaattc**ATGAGCGATACTTATTTGAATC | Amplification of 5’ flanking region of SE1423 (0.5 Kb) |
| 1423-5R | ctatgcgattgaatatacttttcCTTAGCATCCTCTTCATTAAC |  |
| 1423-3F | gttaatgaagaggatgctaaggaAAAGTATATTCAATCGCATAG | Amplification of 3’ flanking region of SE1423 (1.0 Kb) |
| 1423-3R  (SalI) | agct**gtcgac**AGCAGCATACCAATGTCAATC |  |
| 1423-F | CATACGAAGATCGAGGCTAC | Amplification of a partial SE1423 (0.7 Kb) |
| 1423-R | GTACCAACTTGTCCGTCTTG |  |
| DEFB4A | Hs00175474_m1 (TAQMANGene Expression Assays, Applied Biosystems) | Amplification of Beta 4 Defensin |
| S100A7 | Hs00161488_m1 (TAQMANGene Expression Assays, Applied Biosystems) | Amplification of S100 Calcium-binding protein A7 |
| B2M | Hs_00984230_m1 (TAQMANGene Expression Assays, Applied Biosystems) | Amplification of B2 microglobulin, a housekeeping gene |
