# Supplementary material for: Controlling the Growth of the Skin Commensal Staphylococcus epidermidis Using d-Alanine Auxotrophy
Source: mSphere. 2020 Jun 10;5(3):e00360-20. doi: 10.1128/mSphere.00360-20 (PMC7289707; doi:10.1128/mSphere.00360-20)
Supplement: TABLE S3 [file mSphere.00360-20-st003.docx]

**Supplementary Table S3. Growth and survival of SE_ΔΔΔ_ in defibrinated pooled healthy human blood after 24hr incubation**

| **Inoculum at T=0 (CFU/mL)** | **CFU/mL after 24 hr growth** | |
| --- | --- | --- |
|  | SE_ΔΔΔ +_ d-alanine | SE_ΔΔΔ -_ d-alanine |
| 1.0 x 10^0^ | 0 x 10^0^ | 0 |
| 6.0 x 10^2^ | 1.0 x 10^5^ | 0 |
| 2.0 x 10^4^ | 2.0 x 107 | 0 |
| 4.0 x 10^6^ | 1.0 x 10^8^ | 0 |
| 1.4 x 10^8^ | 2.0 x 10^8^ | 1.3 x 10^8^ |

The growth of SE_ΔΔΔ_ was tested in blood +/- 100 µg/mL of d-alanine. Results are the averages of two experimental replicates
